# Mean Airway Pressure—An Informative but Overlooked Indicator of Mechanical Power

**DOI:** 10.1097/CCE.0000000000001305

**Published:** 2025-09-05

**Authors:** Michael G. Michalik, Philip S. Crooke, John J. Marini

**Affiliations:** 1 Division of Pulmonary, Allergy, Critical Care, and Sleep, University of Minnesota, Minneapolis, MN.; 2 Department of Mathematics, Vanderbilt University, Nashville, TN.; 3 Division of Pulmonary, Allergy, Critical Care, and Sleep, University of Minnesota, Minneapolis, MN.

**Keywords:** acute respiratory distress syndrome, artificial respiration, biophysical phenomena, mean airway pressure, mechanical power, ventilator-induced lung injury

## Abstract

Mean airway pressure, a monitored variable continuously available on the modern ventilator, is the pressure measured at the airway opening averaged over the time needed to complete the entire respiratory cycle. Mean airway pressure is well recognized to connect three key physiologic processes in mechanical ventilation: physical stretch, cardiovascular dynamics, and pulmonary gas exchange. Although other parameters currently employed in adults to determine “safe” ventilation are undoubtedly valuable for daily practice, all have limitations for continuous monitoring of ventilation hazard. The purpose of this communication is to explore the often-underappreciated link between mean airway pressure and the mechanical power (cumulative inflation energy/min) that helps determine the adverse consequences of invasive ventilation (ventilator-induced lung injury).

**KEY POINTS**

**Question:** What is the relationship between mean airway pressure and mechanical power in mechanical ventilation?

**Findings:** Mean airway pressure affects physical stretch, vascular dynamics, and pulmonary gas exchange. It is also linked to mean inspiratory pressure, a key component of total inspiratory energy and mechanical power.

**Meaning:** A simple modification of mean airway pressure can be used to estimate mechanical power and may complement the existing indicators of injurious ventilation currently used in clinical practice.

Mean airway pressure ($P_{\bar{aw}}$), a monitored variable continuously available on the modern ventilator, is the pressure measured at the airway opening averaged over the time needed to complete the entire respiratory cycle. Importantly, $P_{\bar{aw}}$ bears a close relationship to the mean alveolar pressure ($P_{\bar{alv}}$) that determines lung and chest wall dimensions (1). While often neglected clinically in adult critical care, $P_{\bar{aw}}$ is routinely used in the neonatal ICU as a component of the oxygenation index, an indicator of the severity of lung injury in neonates (2). The purpose of this communication is to explore the often-underappreciated link between $P_{\bar{aw}}$ and the mechanical power (cumulative inflation energy/min) that helps determine the adverse consequences of invasive ventilation (ventilator-induced lung injury [VILI]).

## DETERMINANTS OF AIRWAY PRESSURE

**Figure 1.**
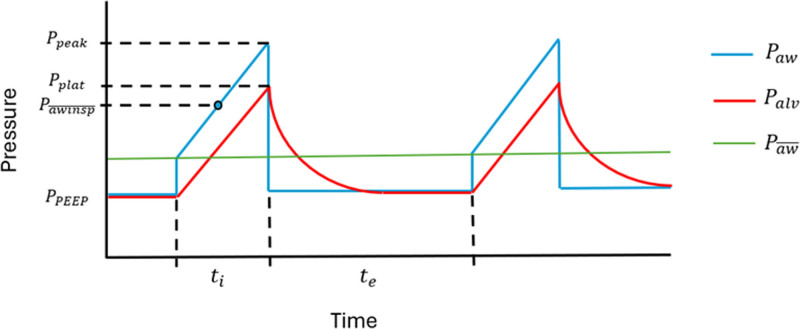
Idealized pressure waveform during passive constant flow ventilation. Mean inspiratory airway pressure ($P_{\bar{awinsp}}$) will occur at exactly ½ of the inspiratory phase of the respiratory cycle. It can, therefore, be used to calculate inflation energy per cycle by simply multiplying it by the tidal volume. Consequently, “power” is the product of inflation energy per cycle and frequency. Palv = alveolar pressure, Paw = airway pressure, $P_{\bar{aw}}$ = mean airway pressure, Ppeak = peak airway pressure, Pplat = plateau pressure, Ppeep = positive end-expiratory pressure, te = expiratory time, ti = inspiratory time.

During both phases of the tidal cycle, the measured airway pressure is composed of the conserved static (positive end-expiratory pressure [PEEP]) and dynamic (driving) “elastic” pressures and the dissipated “resistive” pressure required to push flow through the endotracheal tube and airways. The inspiratory elastic pressure, while necessary to achieve adequate distention and ventilation, can injure parenchymal tissue when a tolerable threshold for lung unit stretch is surpassed (3). Under constant flow, elastic pressure ($P_{alv}$) rises linearly over time during inspiration and decreases exponentially toward PEEP during expiration (**Fig. 1**). As functions of time, both $P_{\bar{aw}}$ and $P_{\bar{alv}}$ are influenced by the ratio of the inspiratory and expiratory durations (I:E ratio). As the I:E ratio lessens (i.e., the expiratory time lengthens with respect to the inspiratory time), $P_{\bar{aw}}$ falls progressively toward PEEP. It follows that apart from tidal volume and PEEP, the flow profile, airway resistance, I:E ratio, and compliance of the respiratory system play roles in determining $P_{\bar{aw}}$ and $P_{\bar{alv}}$.

## LINKING MEAN AIRWAY PRESSURE TO MECHANICAL POWER

Inspiratory mean elastic pressure ($P_{\bar{alvinsp}}$) is a direct determinant of the alveolar portion of the ventilatory “power” as it is currently defined for clinical purposes (4). Ventilatory power, the product of total inflation energy per cycle and frequency, quantifies the cumulative inflation energy delivered per minute and has been linked both to experimental VILI and clinical outcomes (3, 5, 6). Inflation energy per tidal cycle, a force × length relationship, is the product of volume change (area × length) and the pressure that produced it (force/area). During inspiration, total inflation energy is the sum of the component dissipated through airway resistance and the component stored as elastic potential energy that produces tissue stretch. Therefore, the inspiratory energy that stretching requires may be a variable of particular interest to monitor when considering contributors to VILI. Along this line, it is worth noting that under passive conditions, $P_{\bar{aw}}$ (and $P_{\bar{alv}}$) are also key determinants of the loading conditions of both cardiac ventricles and influence pulmonary blood flow distribution. For instance, in the setting of acute respiratory distress syndrome characterized by the reduced ventilation capacity of the functional “baby lung,” a moderate $P_{\bar{aw}}$ may support alveolar recruitment and thereby improve ventilation-perfusion matching (7). However, an excessive $P_{\bar{aw}}$ may redirect vascular flows away from highly compliant (e.g., nondependent) alveoli and toward aerated but vulnerable units with high permeability and thereby promote VILI.

Together with PEEP and the I:E ratio, the $P_{\bar{aw}}$ offers a simple approximation of the mean inspiratory pressure component of total cyclic energy:


$$\begin{array}{l} P_{\bar{awinsp}}=\frac{\left(P_{\bar{aw}}\times \left[t_{i}+t_{e}\right]-P_{PEEP}\times t_{e}\right)}{t_{i}} \end{array}$$

$$\begin{array}{l} or\;\;\;P_{\bar{awinsp}}=\frac{\left(P_{\bar{aw}}\times \left[I+E\right]-P_{PEEP}\times E\right)}{I} \end{array}$$

In these equations, $P_{\bar{awinsp}}$ is the mean inspiratory airway pressure, $t_{i}$ and $t_{e}$ are the inspiratory and expiratory times, respectively, and I and E are the individual components of the I:E ratio. Importantly, as already noted, the product of $P_{\bar{awinsp}}$ and tidal volume tracks the combination of the VILI-focused alveolar component and the dissipated resistive component of inspiratory energy. When multiplied by frequency, inspiratory mechanical power is obtained. This $P_{\bar{awinsp}}$ variable, which is easily automated and displayed, is potentially useful for continuous, noninterventional monitoring of ventilatory energetics without the flow interruption required to record the plateau pressure.

## CONCLUSIONS

In summary, $P_{\bar{aw}}$ connects three separate physiologic processes in mechanical ventilation: physical stretch, cardiovascular dynamics, and mechanical power. Although other parameters that currently are universally employed in adults to determine “safe” ventilation are undoubtedly valuable for daily practice, all have limitations (4). For instance, when used alone, plateau and driving pressures, while influencing the same three physiologic processes as $P_{\bar{aw}}$, do not consider cycling frequency or energetics and depend on interventional stop-flow maneuvers for measurement. Low tidal volume ventilation limits physical stretch but does not account for vascular dynamics or energy load. Mechanical power determined by the original equation of Gattinoni et al (4) (and most simplifying approximations to it), also relies on flow-interrupted measurements and, like ours, includes dissipated energy that seems unlikely to contribute proportionally to VILI. We propose that because $P_{\bar{aw}}$ is available in real-time, holds clear cardiovascular and oxygenation relevance, and with a simple calculation relates more selectively to the mechanical energy of inflation, it complements the panel of indicators currently used in most adult practices.
